# Hippocampal Desynchronization of Functional Connectivity Prior to the Onset of Status Epilepticus in Pilocarpine-Treated Rats

**DOI:** 10.1371/journal.pone.0039763

**Published:** 2012-06-29

**Authors:** Chi-Han Wang, Chou P. Hung, Ming-Teh Chen, Yang-Hsin Shih, Yung-Yang Lin

**Affiliations:** 1 Institute of Physiology, National Yang Ming University, Taipei, Taiwan; 2 Institute of Brain Science, National Yang Ming University, Taipei, Taiwan; 3 Institute of Neuroscience and Brain Research Center, National Yang Ming University, Taipei, Taiwan; 4 Institute of Biophotonics, National Yang Ming University, Taipei, Taiwan; 5 Department of Neurosurgery, National Yang Ming University, Taipei, Taiwan; 6 Department of Neuroscience, Georgetown University, Washington, D.C., United States of America; 7 Laboratory of Neurophysiology, Taipei Veterans General Hospital, Taipei, Taiwan; 8 Department of Neurosurgery, Taipei Veterans General Hospital, Taipei, Taiwan; 9 Department of Neurology, Taipei Veterans General Hospital, Taipei, Taiwan; 10 Department of Neurosurgery, Taipei Veterans General Hospital, Taipei, Taiwan; University of Michigan, United States of America

## Abstract

Status epilepticus (SE), a pro-epileptogenic brain insult in rodent models of temporal lobe epilepsy, is successfully induced by pilocarpine in some, but not all, rats. This study aimed to identify characteristic alterations within the hippocampal neural network prior to the onset of SE. Sixteen microwire electrodes were implanted into the left hippocampus of male Sprague-Dawley rats. After a 7-day recovery period, animal behavior, hippocampal neuronal ensemble activities, and local field potentials (LFP) were recorded before and after an intra-peritoneal injection of pilocarpine (350 mg/kg). The single-neuron firing, population neuronal correlation, and coincident firing between neurons were compared between SE (n = 9) and nonSE rats (n = 12). A significant decrease in the strength of functional connectivity prior to the onset of SE, as measured by changes in coincident spike timing between pairs of hippocampal neurons, was exclusively found in SE rats. However, single-neuron firing and LFP profiles did not show a significant difference between SE and nonSE rats. These results suggest that desynchronization in the functional circuitry of the hippocampus, likely associated with a change in synaptic strength, may serve as an electrophysiological marker prior to SE in pilocarpine-treated rats.

## Introduction

Temporal lobe epilepsy (TLE) is a common type of partial epilepsy, and its development can be triggered by an initial brain damaging insult such as traumatic brain injury, stroke, cerebral tumor, and status epilepticus (SE) [1]. However, the mechanisms underlying epileptogenesis remain largely unknown. In rodents, pilocarpine-induced SE has been commonly used to induce spontaneous recurrent seizures (SRS) and subsequent pathophysiological alterations in the brain comparable to those observed in TLE patients [2], [3], [4], [5]. The duration and magnitude of the initial SE have been thought to be important factors contributing to subsequent development of SRS after a latent period [6], [7], [8]. However, after the application of the same dose of pilocarpine, SE is successfully induced in only a subset of experimental rats [9], [10], [11], [12], which suggests the existence of inter-individual differences in vulnerability to the same excitotoxic insult. It remains unknown whether any difference of neuronal network activity between SE and nonSE rats can be identified before the appearance of SE.

The hippocampal and dentate regions are crucial structures in the development of TLE [13], [14], and neurons in these areas encode information through rate coding (via firing rate) and temporal coding (via modulation of precise spike timing in single neurons and neuronal ensembles) [15]. These codes and neuronal population dynamics are related to precise patterns and strengths of functional connectivity among neurons [16], [17], [18]. Previous studies have analyzed hippocampal unit activity in animal TLE models during interictal [19] or postictal periods [20]. Bower and Buckmaster observed heterogeneous changes in a subset of dentate granule cells few minutes before seizure initiation [21]. In addition to the activities of single neurons, hippocampal networks have also displayed biphasic interictal-to-ictal state transitions during the development of pharmacologically induced epileptic seizures [22]. Therefore, it is interesting to ask whether early changes in hippocampal neural activity following pilocarpine injection are correlated with the occurrence of SE.

We hypothesized that some early patterns in hippocampal neuronal network activity following pilocarpine injection can be identified. In this study, hippocampal neural activity and behavior patterns in rats were measured following administration of pilocarpine. We found a characteristic change in functional connectivity of the hippocampus prior to SE.

## Materials and Methods

### Animals

Twenty-six male Sprague-Dawley rats weighting 200–350 g were used in this study. Each rat was individually housed in a heat-regulated environment (12-h day/night cycle) with food available *ad libitum*. All procedures were approved by the Institutional Animal Care and Use Committee of National Yang-Ming University, Taipei, Taiwan, and adhered to the United States National Institutes of Health (NIH) Guidelines for the Care and Use of Laboratory Animals.

### Surgical Implantation of Electrodes

Rats were anesthetized with isoflurane (3–5%). A microwire array consisting of 16 Teflon-insulated stainless steel electrodes (AM systems, Carlsborg, WA; #790700) was used with an electrode-diameter of 50 μm and an inter-electrode separation of 250 μm [23]. The microwire array was surgically implanted into the left hippocampus (AP -3.9 mm; ML +2.4 mm; DV -3.7 mm), and a reference electrode was placed in the cerebellum [24]. Four screws were attached to the skull to help anchor the dental cement.

### Experimental Protocol

**Figure 1:**
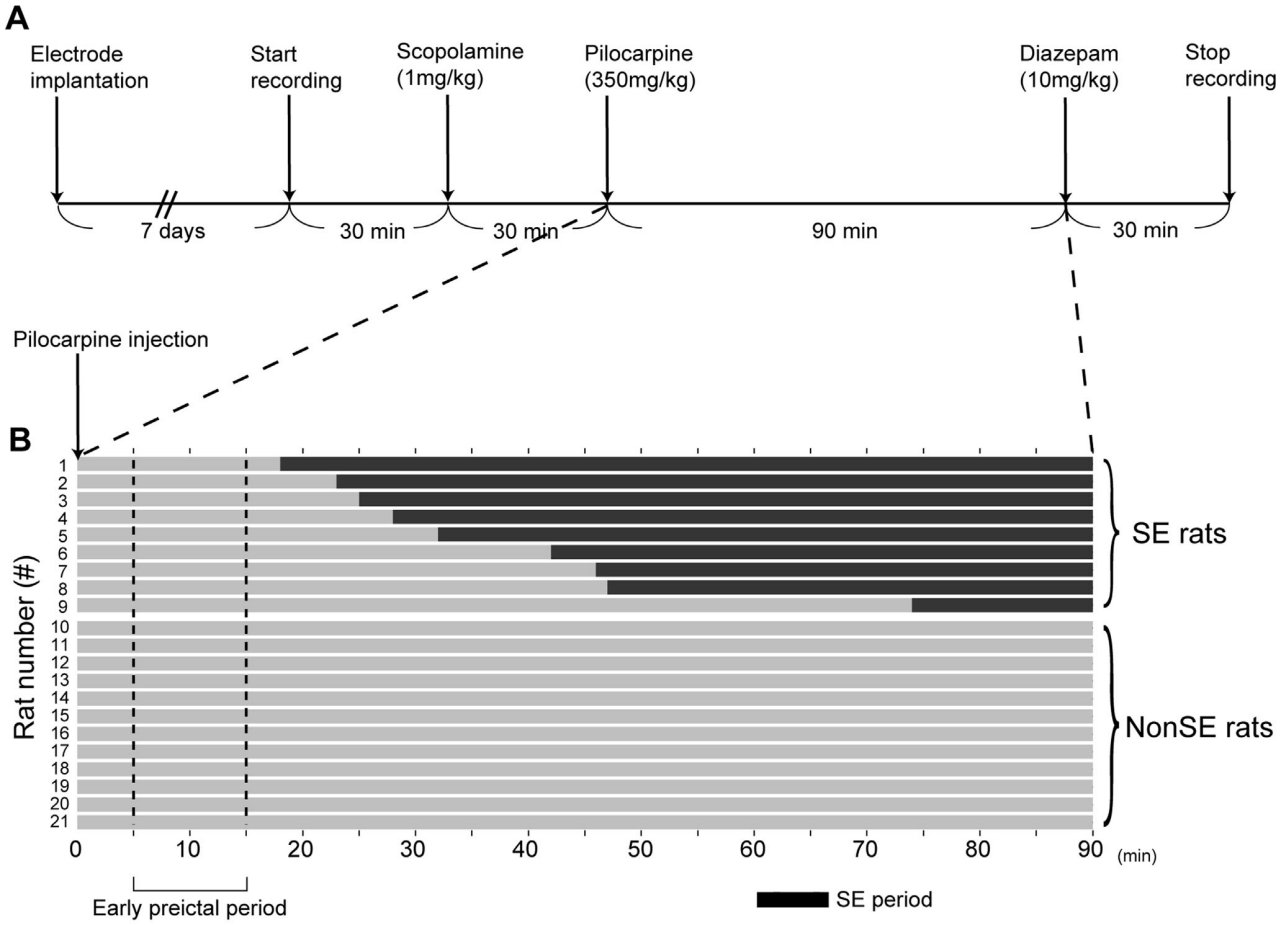
Outline of the experimental procedure. **A**. Experimental steps for induction of status epilepticus (SE). **B**. Temporal profiles of SE in 9 rats after injection of pilocarpine. No SE was identified in 12 rats. The black bar indicates the occurrence of SE. The early post-pilocarpine period was defined as the time period of 5–15 min after injection of pilocarpine, as delimited between the two vertical dashed lines.

After 7 days of recovery from surgery, the microwire array of electrodes was connected to the recording apparatus via a head stage. After a 30-minute baseline recording, a dose of methyl-scopolamine (1 mg/kg i.p., Sigma) was administered, followed 30 minutes later by an injection of pilocarpine (350 mg/kg i.p., P 6503, Sigma). At 90 minutes after pilocarpine injection, diazepam (10 mg/kg i.p., Dupin injection, China Medical) was given to alleviate seizure severity. Experimental procedures are shown in Fig. 1A.

### Behavioral Observations

To detect SE, we simultaneously recorded LFP and behavior. An electrographic activity of a seizure was defined as a high-amplitude (2 times higher than the baseline noise), high-frequency (>5 Hz) discharge that lasted at least 10 seconds. Rat behavior was recorded by a video camera (KMS-D12IR-8, ksounds), and seizure patterns were assessed according to the five-stage classification of Racine [25]. In this study, SE was defined as a continuous motor seizure at stages 4-5 with a concomitant electrographic seizure activity. The rats were divided into SE and nonSE groups according to the presence or absence of SE during the 90-minute recording period following an injection of pilocarpine (Fig. 1B).

### Confirmation of Electrode Positions

**Figure 2:**
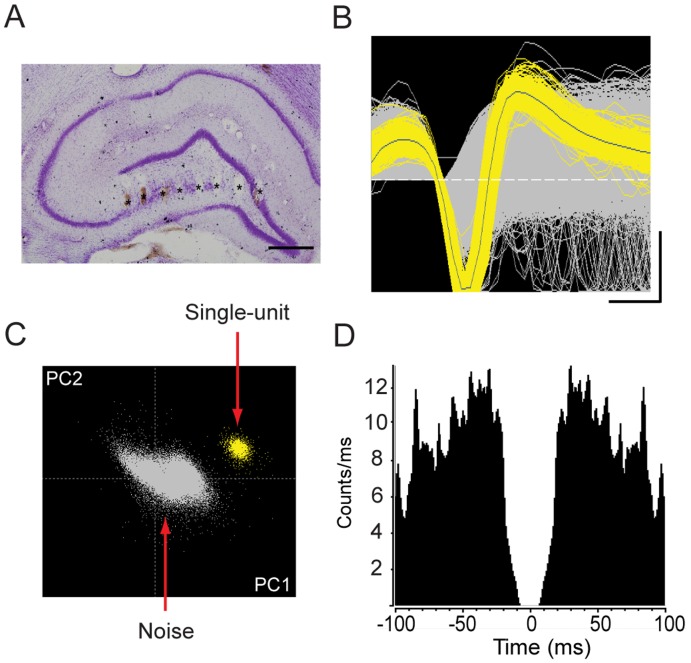
Recording and analysis profiles of neuronal ensembles. **A**. Histological locations (asterisks) of recording electrode tips in the hippocampus. Scale bar indicates 500 μm. **B**. Waveforms of hippocampal spikes (yellow) and noise signals (whitish-gray) separated by morphology (calibration: 0.2 ms, 100 μV). **C**. Clusters of single units and noise signals separated according to the first two principal components (PC1 on x-axis; PC2 on y-axis). **D**. Autocorrelogram of a single unit with an absolute refractory period of a minimum of 2 ms.

At the end of the experiment, rats were processed to confirm the location of the microwire electrodes. Rats were deeply anesthetized with chloral hydrate (500 mg/kg) and a 10-microampere positive current was applied for 10 seconds to deposit Fe^2+^ into the tissue [26]. The rats were perfused with 4% paraformaldehyde solution followed by a 3% potassium ferrocyanide/4% paraformaldehyde solution. Extracted brain tissue was washed with 0.1 M phosphate buffer and was treated with the same fixatives overnight, followed by treatment with 30% sucrose in phosphate buffer at 4°C for the next two nights. Brain tissue was sliced coronally at 50 μm, and electrode tip positions were identified by blue marks in the tissue created by the reaction products of the iron deposition. Sections containing blue marks were counterstained with cresyl violet (Nissl staining, Fig. 2A).

### Recordings of Spike and LFP Signals

We recorded LFP and neuronal ensemble activity from the hippocampus using a 16-channel neuronal recording system (Plexon Inc., Dallas, TX). LFP signals were preamplified (1000 x), filtered (0.5–100 Hz, 1-pole Butterworth filter), and digitized at 1 kHz. Spike activities were bandpass-filtered between 150 Hz and 8 kHz (3-pole Butterworth filter) and were digitized at 40 kHz. The spike signals were imported into NeuroExplorer (Plexon, Inc.) for subsequent offline analysis. All extracted waveforms were sorted by amplitude and shape (Fig. 2B). Based on principal component analysis (PCA), all waveforms were projected into the first two principal components for unit separation (Fig. 2C). We used the putative single units with a minimum absolute refractory period of 2 ms in the autocorrelogram (Fig. 2D) for further analysis.

### Monitoring of SRS

Animals were monitored with a video camera for SRS for 12 h/week (2 h/day, 7 days/week) from Weeks 2 to 5 after pilocarpine injection. The SRS was classified according to Racine’s scale [25].

### Data Analysis

We calculated the mean firing rate within 30 minutes before and after pilocarpine treatment in 60-sec bins and plotted the continuous firing rate change. To determine the firing rate change prior to the onset of SE, the neuronal rates before pilocarpine injection [-10∶0 minutes] were compared with the rates after pilocarpine injection [5∶15 minutes].

To evaluate the strength of functional connections, we measured the relative spike times of all neuronal pairs using cross-correlation analysis [27], [28], [29]. We began by computing the raw cross-correlation histograms (CCH_raw_) for pairs with at least 1500 spikes per spike train. The histogram was computed by counting the relative spike times between two spike trains, binned at 1 ms and normalized by the square root of the product of the spike number in cell 1 multiplied by the spike number in cell 2 (N_spikes in cell 1_ x N_spikes in cell 2_)^0.5^. We then separated the raw cross-correlation histogram into two parts. One part, based on coincident activity slower than 50 ms (JITTER_50_, calculated via the ‘jitter’ method of Kohn and Smith [28]), is driven by common inputs, slow covariations, and possible changes in animal behavior. The other part, based on coincident activity faster than 50 ms (COIN_50_ =  CCH_raw_ – JITTER_50_), has been considered an index of functional connectivity between pairs of neurons and may reflect synaptic strength. COIN_50_ is also referred to as the cross-correlogram (‘CCG’). The CCH_raw_, JITTER_50_, and histograms were smoothed with a 7-ms boxcar window. To determine pairs with significant peaks, we randomly shuffled segments of activity while preserving the average rate. The results from -150 to 150 ms were examined, and significant pairs were defined when the CCH_raw_ peak exceeded the 5-95% envelope of JITTER_50_ of 2020 jittered spike trains in the -50 ms to 50 ms window (101 bins/5% = 2020). We calculated the connection probability (percentage of significant pairs) and measured the change in CCG peak height following pilocarpine injection in SE and nonSE groups.

### Statistical Analysis

In this study, all values are reported as mean ± the standard error of the mean (SEM), and p<0.05 was considered as statistically significant. To tell the difference between SE and nonSE rats, we compared the changes in connection probability with Chi square tests and evaluated the changes in connection strength and peak width with two-sample t-tests. Paired t-tests were used to assess the difference of neuronal firing rates between pre- and post-pilocarpine treatment in the same rat.

## Results

### Pilocarpine-induced SE

Within 90 minutes following pilocarpine injection, 14 of the 26 rats showed stages 4-5 SE. Five of the 14 rats were excluded from further analysis because of either losing the headset (n = 1) or dying during severe convulsions (n = 4). Therefore, a total of 9 SE rats were used for subsequent analysis. Twelve rats were defined as nonSE rats because no sustained motor seizures were identified throughout the recording period (Fig. 1B).

**Figure 3:**
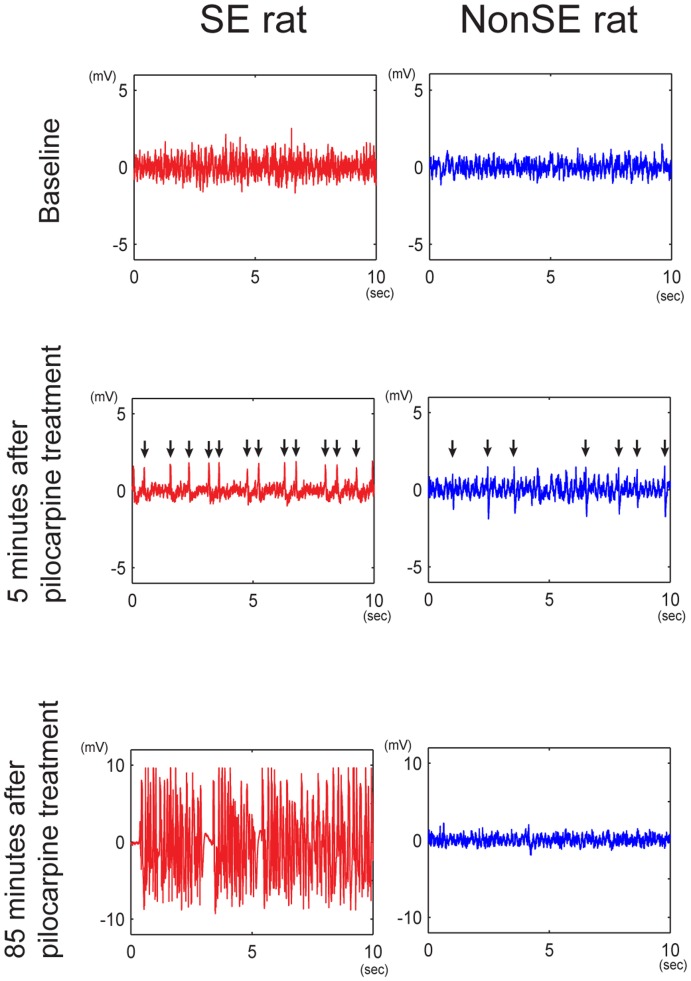
Local field potentials in 10-sec time windows of representative SE and nonSE rats at baseline period (upper panel) and at 5 min (middle panel) and 85 min (lower panel) after pilocarpine treatment.

Abnormal behavior patterns, including head nodding, automatisms, and limb shaking were observed about one minute after pilocarpine injection in all rats (1.0±0.2 minutes for SE group and 1.1±0.2 minutes for nonSE group, p = 0.65). In SE rats, sustained stages 4-5 seizures began 37.5±5.8 minutes after pilocarpine injection. The LFP was recorded for 90 minutes following pilocarpine injection to show the electrographic patterns in SE and nonSE rats (Fig. 3). The seizure and LFP patterns showed similar temporal profiles with respect to the occurrence of SE (Table 1).

**Table 1:** Mean onset time (± SEM) of status epilepticus (SE) relative to behavioral and local field potential (LFP) patterns after i.p. pilocarpine injection in nonSE and SE groups.

		SE group (minute)	NonSE group (minute)
Behavior	Onset of intermittent convulsions	1.0±0.2	1.1±0.2
	Onset of stages 4-5 seizures	37.5±5.8	No sustained seizures
LFP	Onset of repetitive isolated spikes	2.6±0.5	3.6±0.7
	Onset of almost continuous discharges	30.6±3.5	No continuous discharges

### Firing Rate Changes in SE and nonSE Rats

**Figure 4:**
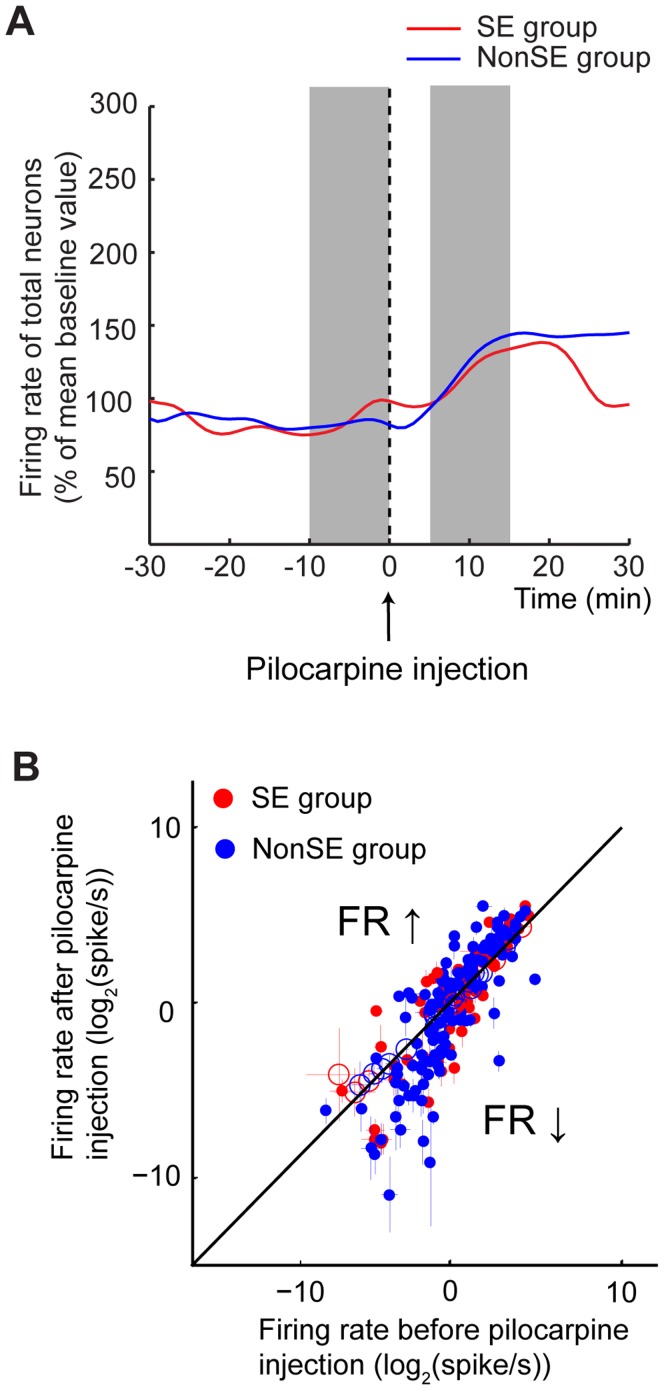
Changes in the firing rates of hippocampal units in pilocarpine-treated rats. **A**. Relative discharge rates 30 min before and after pilocarpine treatment in SE (red) and nonSE (blue) rats. **B**. Rate-by-rate comparison of firing rate change before [-10∶0 minute] and after [5∶15 minute] pilocarpine injection (see gray zones in panel A) of individual neurons from SE (red dots) and nonSE (blue dots) rats. Filled circles indicate significant difference of firing rate after pilocarpine injection. Open circles indicate no change in firing rate.

In this study, a total of 262 units were recorded in 21 rats (7.7±1.6 units/rat). Figure 4A shows the averaged time-varying firing rates of SE (red) and nonSE (blue) rats. An increase in firing rate following pilocarpine injection was found in both SE (from 3.5±0.6 Hz to 4.8±0.9 Hz, p = 0.0067) and nonSE rats (from 2.5±0.3 Hz to 3.8±0.5 Hz, p = 0.002). The firing ratios relative to the baseline level were 1.4±0.2 and 1.4±0.1 for SE and nonSE groups (p = 0.91), respectively. As shown in Fig. 4B, some units (solid points) showed an increased firing rate after pilocarpine injection, while the others exhibited either a decrease or no change in firing rate.

### Differences in Functional Connectivity Between SE and nonSE Rats

**Figure 5:**
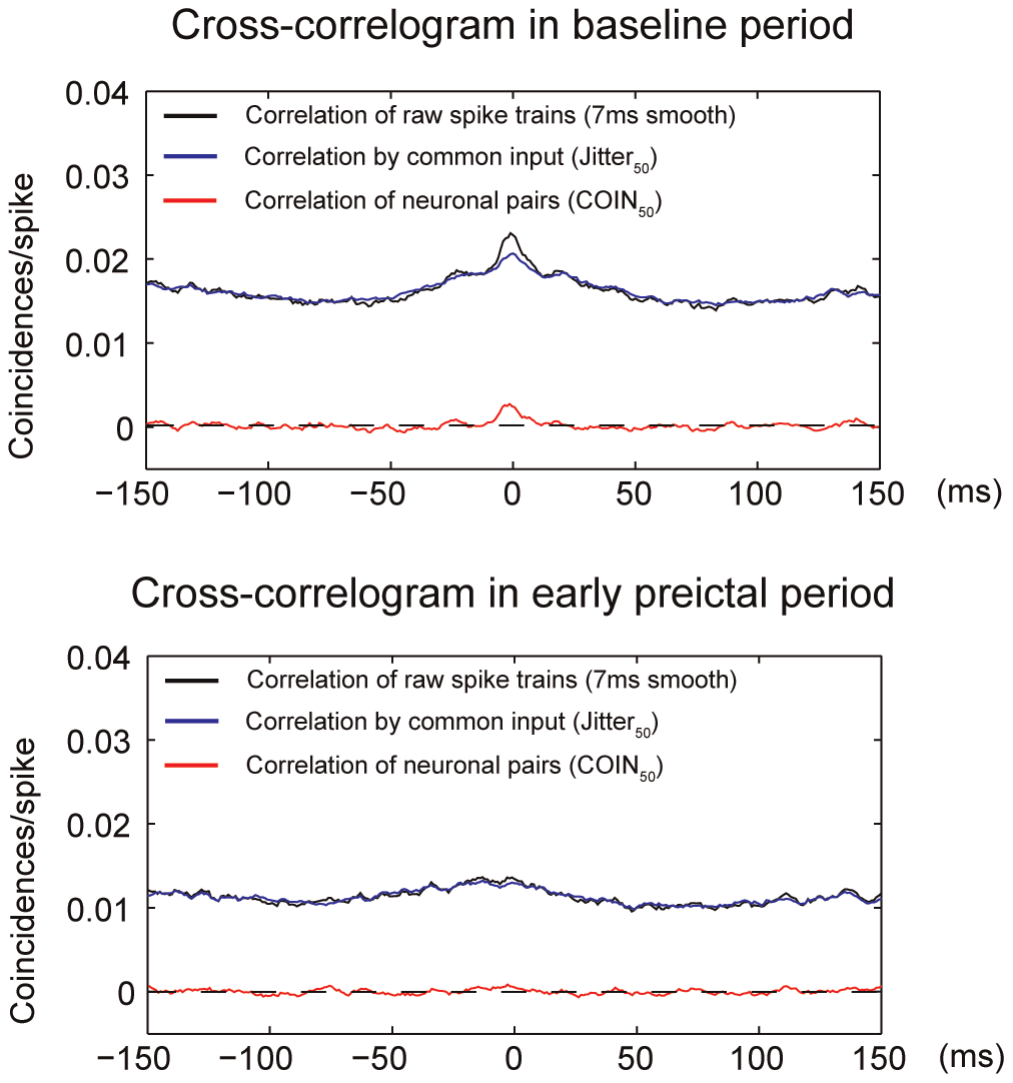
Cross-correlation histograms show coincident firing of a sample pair of neurons from one SE rat between the baseline period and early preictal period. Black line indicates the raw correlation smoothed with a 7 ms boxcar window (CCH_raw_). The jitter-predicted correlation (Jitter_50_, blue) indicates the correlation driven by common input, as determined by jitter shuffling of the spike train with a 50 ms window. The cross-correlogram (‘CCG’, or COIN_50_, red) corresponds to pair-specific coincident activity, defined as the difference between raw coincidences and jitter coincidences.

We used cross-correlation analysis to measure functional connectivity in 80 SE pairs and 86 nonSE pairs. We defined the strength of functional connectivity as the peak height of the cross-correlation histogram (CCH_raw_, see methods). Figure 5 shows cross-correlation histograms from an example pair during baseline (Fig. 5A) and early preictal periods (Fig. 5B). Because synchronous activity might arise from both common input and direct connections, we used a jitter correction method (50 ms window) to separate CCH_raw_ (black) into the correlation by slow common input (‘Jitter_50_’, blue) and fast coincident activity between neuronal pairs (‘COIN_50_’, red) [28]. The common input component (Jitter_50_) indicates the portion of the synchronous activity driven by slow (>50 ms) correlated changes in firing rate, which suggests a large-scale change in correlated activity. Conversely, a change in synaptic efficacy between two neurons should be reflected as a change in fast coincident activity (COIN_50_), although some changes in network synchrony may also occur at fast time scales.

**Figure 6:**
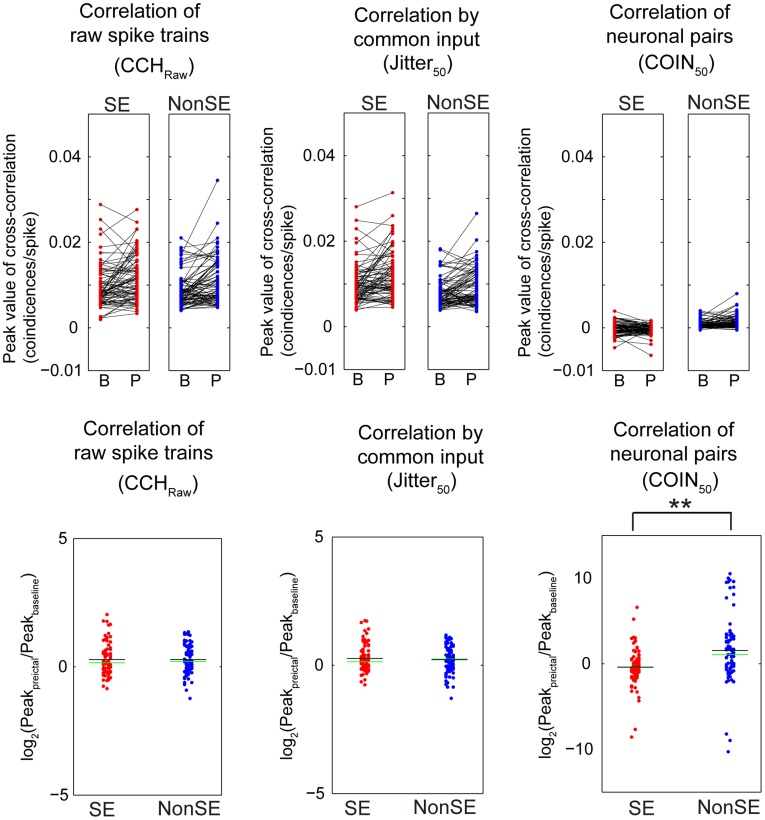
Changes in functional connectivity across all neuronal pairs from cross-correlation analysis. Upper panel shows the raw correlation (CCH_raw_), correlation driven by common input (Jitter_50_), and correlation of neuronal pairs (COIN_50_) based on peak values at baseline and early preictal periods. Lower panel shows log ratios of the coincident peak of early preictal period to that of baseline period. The vertical scatter plots integrate the distributions of all ratio results in CCH_raw_, Jitter_50_, and COIN_50_. Each data point represents a result from one neuronal pair. The mean peak ratio is indicated by black horizontal line, and the median peak ratio is shown in green. The correlation value of neuronal pairs was significantly larger in nonSE rats than SE rats.

Next, we compared changes in peak height within the same pair (upper panel in Fig. 6). The values of peak height from all neuronal pairs are plotted in vertical scatter plots for SE (red points) and nonSE (blue points) pairs. The black lines show the change in peak height before and after pilocarpine injection. The lower panel of Figure 6 shows the log values of peak height change from baseline to post-pilocarpine pre-ictal period. With raw correlations (CCH_raw_), an increase in peak height after pilocarpine administration was found in both SE and nonSE pairs (SE and nonSE: 121.7±4.8% and 121.8±4.4%, respectively; p = 0.98, T_83, 79_ = 0.0196). Pilocarpine increased the correlation by common input (JITTER_50_) in both SE and nonSE rats (120.2±4.3% and 116.3±4.1%, respectively; p = 0.58, T_83, 79_ =  −0.5527). Notably, fast coincident activity between neuronal pairs (COIN_50_) was decreased in SE rats and was increased in nonSE rats (76.0±18.7% and 194.2±33.8%, respectively; p = 1.12×10^−4^, T_83, 79_ = 4.0082).

Thus, following pilocarpine injection, there was an overall increase in synchronous activity and common input in both SE and nonSE rats, but fast coincident activity showed opposite changes for SE and nonSE rats. This difference between common input and coincident activity was significantly larger in SE than in nonSE pairs (χ^^2^^_1_ = 26.59, p = 2.51×10^−7^ vs. χ^^2^^_1_ = 0.05, p = 0.82, based on the number of pairs showing increases in peak height). Thus, SE and nonSE rats exhibited a significant difference in network synchrony at the very early period before SE.

### SRS During Weeks 2-5 After Pilocarpine Injection

In the 9 SE rats, 5 rats died during week 1, and the other 4 rats exhibited SRS at approximately day 9 (9.5±1.9 days) after pilocarpine injection. However, no SRS event was identified in the 12 nonSE rats.

## Discussion

This study focused on hippocampal activity in the early pre-SE period of pilocarpine-treated rats. The decrease in coincident activity prior to SE suggests desynchronization of functional connectivity of neuronal pairs is an early electrophysiological feature of SE rats. In contrast, a characteristic early pattern preceding SE was not found in conventional EEG parameters such as neuronal ensemble activity and LFP signals.

Electrophysiological changes during preictal, ictal and postictal periods have been analyzed in previous studies. Heterogeneous firing rate and emerged discharge in the hippocampus have been observed in the preictal period [21], [30]. Ictal spreading was passed through hippocampal connections [31], [32]. During the latent period, interictal spikes disrupted normal hippocampal function [20]. However, the neuronal characteristics in the hippocampus before the onset of SE are still uncertain. Here, we identified the decreased strength of coincident firing in SE neurons which indicates desynchronization of functional connectivity in the hippocampus prior to the appearance of SE in pilocarpine-treated rats. Notably, this desynchronization was found exclusively in SE rats, but the mechanisms underlying this decreased functional connectivity in the hippocampus remain largely unknown. In line with previous studies [33], [34], the alteration of functional connectivity preceding SE identified in this study may reflect a dysregulation phenomenon in the hippocampal and subicular/entorhinal networks. Our data suggest that desynchronization of hippocampus may render brain circuits to become more vulnerable to an excitotoxic stimulation.

Functional changes in the brain can be estimated with various parameters, such as synchronized oscillations of different frequency bands, signal correlations in neuronal firing and LFP, and coincident discharges in single units [35], [36], [37], [38]. For the coincident discharge, the raw correlation in one pair is composed of the correlation of the neuronal pair and the correlation by common input. To demonstrate the correlation of neuronal pairs, the correlation by common input should be excluded since it is likely driven by a pool of cortical neurons providing inputs to the recorded pair. Conventional cross-correlation algorithms for measurement of spike timings and coincident firing do not extract the information of functional connectivity embedded in common input. To focus on the pure functional connectivity of hippocampal neurons, we modified the cross-correlation algorithm with a jitter correction and COIN_50_ in functional connectivity analysis [28]. With this modification, the interference of common input from different regions can be removed. Our data suggests that disturbances of neuronal correlation, network dynamics and disconnected synchrony precede the development of prominent seizures, which is in agreement with previous studies [22], [37], [39]. However, the effect of low firing rate neurons (<2.5 Hz) were excluded because only the spike trains with >1500 spikes in 10-min recording had sufficient spikes for statistical analysis. Direct patch-clamp recordings from hippocampal neuron pairs in future studies will help elucidate the mechanisms underlying the connectivity change prior to SE.

In this study, 54% of pilocarpine-treated rats exhibited SE, but the rest (46%) did not develop SE. The heterogeneous responses of rats to the same dose of pilocarpine have been found in previous studies [9], [10], [11], [12]. According to our follow-up evaluation at Weeks 2-5, the likelihood of developing SRS is higher in SE rats compared with nonSE rats. Further studies are warranted to clarify the role of the hippocampal connectivity change prior to SE in the development of SRS. However, there are also potential limitations in the present study. First, the occurrence of SRS might be underestimated because behavior was recorded by intervals but not by continuous observations. Second, the connectivity pattern within the hippocampus might not reflect the only change to the neuronal network, because the hippocampal area is not necessarily the only site of seizure onset in a pilocarpine SE model [21]. Further studies with simultaneous recordings in different regions may help clarify the neuronal correlates of these circuit changes.

Previous studies have identified neuronal complexity loss, a decrease of dynamical similarity, and increases in accumulated energy from scalp EEG recordings before the seizure attack [40]. One recent human study has observed firing rate heterogeneity from the middle frontal and temporal gyri before and during seizure events, and the probability of spiking provides one possible algorithm for seizure prediction [41]. In the present study, mean firing rates of all units gradually increased after pilocarpine injection, regardless of whether or not SE is induced, but heterogeneous firing of individual neurons were found. The findings suggest that a brain insult by pilocarpine gave rise to hyperexcitability in hippocampal neurons, but the development of SE may depend on a substantial change in functional connectivity.

In conclusion, the hippocampal desynchronization reflected as a change of firing coincidence between neurons may be a useful biomarker for predicting the subsequent occurrence of SE. Further studies are needed to clarify the role of the early functional connectivity change in the development of SRS.
